# The Complex Genetic Architecture of the Metabolome

**DOI:** 10.1371/journal.pgen.1001198

**Published:** 2010-11-04

**Authors:** Eva K. F. Chan, Heather C. Rowe, Bjarne G. Hansen, Daniel J. Kliebenstein

**Affiliations:** 1 Department of Plant Sciences, University of California Davis, Davis, California, United States of America; 2 Department of Plant Biology and Biotechnology, Copenhagen University, Copenhagen, Denmark; The University of North Carolina at Chapel Hill, United States of America

## Abstract

Discovering links between the genotype of an organism and its metabolite levels can increase our understanding of metabolism, its controls, and the indirect effects of metabolism on other quantitative traits. Recent technological advances in both DNA sequencing and metabolite profiling allow the use of broad-spectrum, untargeted metabolite profiling to generate phenotypic data for genome-wide association studies that investigate quantitative genetic control of metabolism within species. We conducted a genome-wide association study of natural variation in plant metabolism using the results of untargeted metabolite analyses performed on a collection of wild *Arabidopsis thaliana* accessions. Testing 327 metabolites against >200,000 single nucleotide polymorphisms identified numerous genotype–metabolite associations distributed non-randomly within the genome. These clusters of genotype–metabolite associations (hotspots) included regions of the *A. thaliana* genome previously identified as subject to recent strong positive selection (selective sweeps) and regions showing trans-linkage to these putative sweeps, suggesting that these selective forces have impacted genome-wide control of *A. thaliana* metabolism. Comparing the metabolic variation detected within this collection of wild accessions to a laboratory-derived population of recombinant inbred lines (derived from two of the accessions used in this study) showed that the higher level of genetic variation present within the wild accessions did not correspond to higher variance in metabolic phenotypes, suggesting that evolutionary constraints limit metabolic variation. While a major goal of genome-wide association studies is to develop catalogues of intraspecific variation, the results of multiple independent experiments performed for this study showed that the genotype–metabolite associations identified are sensitive to environmental fluctuations. Thus, studies of intraspecific variation conducted via genome-wide association will require analyses of genotype by environment interaction. Interestingly, the network structure of metabolite linkages was also sensitive to environmental differences, suggesting that key aspects of network architecture are malleable.

## Author Summary

Understanding how genetic variation can control phenotypic variation is a fundamental goal of modern biology. We combined genome-wide association mapping with metabolomics in the plant *Arabidopsis thaliana* to explore how species-wide genetic variation controls metabolism. We identified numerous naturally-variable genes that may influence plant metabolism, often clustering in “hotspots.” These hotspots were proximal to selective sweeps, regions of the genome showing decreased diversity possibly from a strong selective advantage of specific variants within the region. This suggests that metabolism may be connected to the selective advantage. Interestingly, metabolite variation in wild Arabidopsis is highly constrained despite the significant genetic variation, thus providing the plant un-sampled metabolic space if the environment shifts. The observed structuring of genetic and metabolic variation suggests individual convergence upon similar phenotypes via different genotypes, possibly intra-specific parallel evolution. This phenotypic convergence couples with a pattern of genotype—phenotype association consistent with metabolite variation largely controlled by numerous small effect genetic variants. This supports the supposition that large magnitude variation is likely unstable in a complex and interconnected metabolism. If this pattern proves generally applicable to other species, it could present a significant hurdle to identifying genes controlling metabolic trait variation via genome-wide association studies.

## Introduction

There is a direct link between the genotype of an organism and its metabolite levels, which can subsequently impact other quantitative traits [1]–[5]. Traditionally, studies of metabolic traits and their genetics have focused on small numbers of metabolites. However, recent technological advances have enabled broad-spectrum, untargeted metabolite profiling, or metabolomics [6]. Variation in the levels of metabolites within a species or population is largely quantitative, moderately heritable [7]–[10], and shows polygenic inheritance [11]– controlled by the interaction of environmental and genetic factors [13]–[14].

To better understand the genetic control of metabolite abundance, linkage disequilibrium analyses, or quantitative trait locus (QTL) mapping, using structured populations are generally performed (e.g. [7], [9]–[10], [15]). While structured populations may contain significant fractions of the variation present within a species, their use is disadvantaged by the limited number of recombination. Genome-wide association (GWA) mapping provides a complementary approach to QTL-mapping as it allows wider sampling of the genotypes present within a species. GWA seeks to associate phenotypes with genotypes, at a genome-wide level, using ‘unrelated’ individuals [16]. However this increase in genotypic sampling can be negated by a lack of corresponding increase in phenotypic diversity that can consequently lead to an increase in detection of both false-positive and false-negative genotype-phenotype associations [17]–[19]. A limitation to both QTL-mapping and GWA is the generation of false positives within regions of high linkage disequilibrium (also known as ghost QTL), which can conversely be turned into false negatives when one errs too much on the side of caution and ignores the possibility of multiple distinct but co-localized causative polymorphisms [17]–[20].

In addition to the genetic-metabolite relationships, the study of metabolite-metabolite correlation can also enhance understanding of cellular processes as inter-metabolite correlations are expected to reflect underlying biochemical networks [21]–[23] . If biochemical networks are coordinately controlled by common genetic determinants, then one may expect similar loci identified by GWA to influence the accumulation of groups of metabolites within a biochemical network. Thus far, metabolic networks have generally been reconstructed using covariance of metabolites across replicated measures [24], whereas covariance of metabolites across different genetic backgrounds have not been examined. Equally, little is known about the stability of metabolic networks under different environmental conditions. Metabolite levels are sensitive to numerous environmental inputs and as such, the genetic control of metabolite levels may also be affected by the organism's environment [25]–[32].

To investigate the natural genetic diversity underlying a plant metabolome, we obtained broad-spectrum metabolite profiles from a densely-genotyped collection of 96 naturally occurring (wild) *A. thaliana* accessions chosen to represent the geographic distribution and genetic diversity of the species. These metabolite levels were used as phenotypic traits in a GWA analysis and for the reconstruction of metabolic networks. To query the genotypic component controlling diversity within the *A. thaliana* metabolome, the metabolite profiles of 96 accessions were measured twice from tissues collected from two independent trials. Between 70–75% of metabolites measured in our analyses were detected in both trials, though 70% of these were found at different levels. Our network analyses identified few significant metabolite-metabolite correlations, indicating minimal direct interaction between metabolites, and few common metabolite-metabolite correlations between the two trials, suggesting a strong environmental component to the architecture of metabolic networks. Finally, we confirmed that metabolite abundance is heritable and under polygenic control, and further showed that metabolites are under pleiotopic control where few genomic regions were associated with changes in levels of tens to hundreds of metabolites.

## Results

### The metabolome is quantitatively dynamic

To study both the natural phenotypic and genetic variation in the *A. thaliana* metabolome, we identified and measured metabolite levels, using non-targeted GC-TOF-MS, in leaf tissues of 96 *A. thaliana* accessions harvested with replication in two separate experiments. One experimental set of plants was harvested early in the plants' photoperiod (Experiment A) and the other late in the photoperiod (Experiment B); all other environmental (e.g. growth chamber condition) and technical (method of planting and growth) variations were minimized. A total of 327 plant metabolites were detected and present in >50% of the samples (see M&M for preprocessing steps). Of these, 194 were detected in both experiments and 133 were unique to one of the two experiments. Of the 194 metabolites detected in both experiments, the majority (133 = 69%) accumulated to significantly different levels between experiments (t-tests Bonferroni-adjusted P<0.05; Table S1).

**Figure 1:**
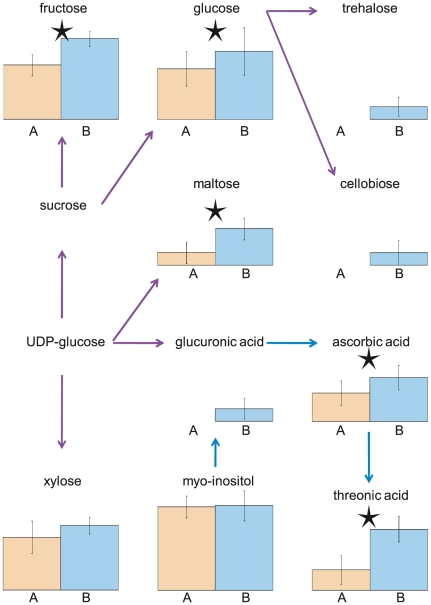
Metabolites in the Starch and Ascorbate pathways are more abundant in Expt. B (collected at PM) compared to Expt. A (collected at AM). Shown are the average metabolite levels across 96 accessions in Expt. A (orange) and Expt. B (blue). Error bars denote one standard deviation and asterisks denote significant and greater than two-fold difference between experiments. Purple arrows denote starch and sucrose metabolic pathway members and blue arrows correspond to the ascorbate metabolic pathway.

While the two experiments were not conducted concurrently, the plants were grown in the same chambers using as nearly identical conditions as possible. As such, we expect that the time of harvest to be the major cause of variation between the two experiments. Supporting this, the identity of several of the differentially abundant metabolites is consistent with photoperiod-sensitive accumulation. For example, phytol, a constituent of chlorophyll that mainly functions to absorb and transfer light energy, was 6-times more abundant in the AM samples (Expt. A) compared to the PM samples (Expt. B) [33]. Conversely, many metabolites were present at higher levels in Experiment B relative to Experiment A, including metabolites involved in starch (e.g. fructose, maltose, glucose) and ascorbate (e.g. ascorbate, threonic acid) metabolism as expected (Figure 1; [34], [35]). These differences between the experiments, however, appear to be largely metabolite specific as no metabolic class or pathway was over or under represented among differentially abundant metabolites (data not shown). While the data suggests that time of day for harvest is the major difference, it is not inherently the only difference between the two experiments.

### The metabolome is qualitatively dynamic

The observed difference in the type and amount of metabolites present in the two experiments could result from 1) a common metabolic network being differentially regulated under the two conditions or 2) differences in the structure of metabolite relationships between the two experiments. To investigate these hypotheses, we constructed metabolite correlation networks across genotypes which were used as surrogates for metabolic networks. If differential metabolite accumulation resulted from differential regulation of a common metabolic network, we would expect similar correlation structure and network properties between experiments, involving different metabolites. This would be akin to being given two different low resolution images and testing if they represent the same object. The pixels (metabolites) presented may differ in each image but still conveys the same object (network). If instead, the structure of the network also differs between the experiments, then the correlation structure will fundamentally differ between the experiments.

**Figure 2:**
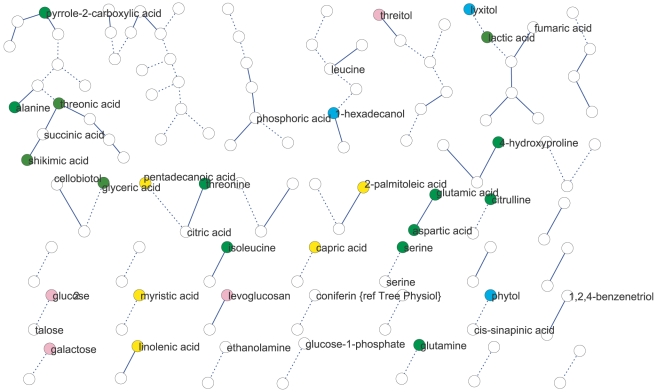
Metabolic network for Expt. A generated from significant (local FDR<20%) partial correlations between metabolites. Metabolites are represented by nodes (circles) and significant metabolite-metabolite connections are represented by edges (lines). Solid lines define positive connection and dotted lines indicated negative correlations. Colors of circles indicate: amino acids (bright green), fatty acids (yellow), carbohydrates (pink), hydroxy acids (dark green), alcohols and polyols (blue).

**Figure 3:**
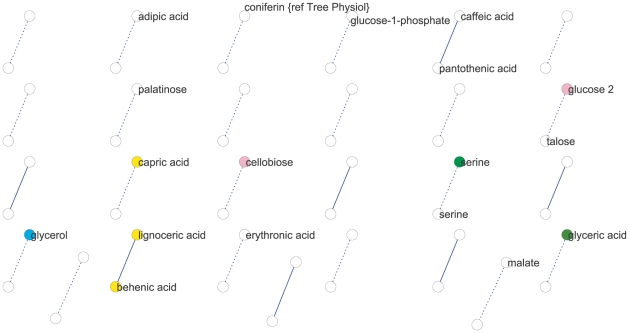
Metabolic network for Expt. B generated from significant (local FDR<20%) partial correlations between metabolites. Metabolites are represented by nodes (circles) and significant metabolite-metabolite connections are represented by edges (lines). Solid lines define positive connection and dotted lines indicated negative correlations. Colors of circles indicate: amino acids (bright green), fatty acids (yellow), carbohydrates (pink), hydroxy acids (dark green), alcohols and polyols (blue).

We used partial (first-order) correlation to analyze the genetically identified metabolic network [36]–[37]. This approach was chosen over more common, zero-order correlation methods, such as Spearman's correlation, because first-order correlations account for indirect correlations, a problem that can inflate clustering coefficients and subsequently lead to false network cliquishness [38]. Strong indirect correlation within *A. thaliana* metabolism have previously been found using both growth and starch accumulation as the likely cause of these indirect correlations, supporting our use of first-order correlations [39]–[40]. Few significant correlations among metabolites were identified: at a local FDR of 5%, only 30 correlations between 52 metabolites (Expt. A) and 17 correlations between 34 metabolites (Expt. B) were observed. This lack of significant correlation between pairs of metabolites implies that their levels were not coordinated in the 96 genetic backgrounds used. Increasing the local FDR to 20% as suggested by [36] resulted in 89 significant correlations of 126 metabolites (Expt. A) and 27 significant correlations of 54 metabolites (Expt. B) (Figure 2 and Figure 3). Even at this more lenient threshold, connectivity is much lower than that observed using Spearman's ρ correlation (Figures S1, S2, S3, S4), suggesting few true direct correlations. In all, only six pairs of metabolite-metabolite correlations were identified in both the Expt. A and Expt. B genetic networks, suggesting each pair of these metabolites are genetically linked under these two conditions. Interestingly, these six consistent metabolite-metabolite connections all represented negative correlations. Two serine compounds likely linked by serine racemase [41] (L-serine (213294) & D-serine(227962) [42]–[43]) showed strong negative correlations in both experiments. The other correlated metabolite-pairs were glucose/talose, glucose-1-phosphate/227973, 208686/216838, 200622/215682, 226280/228078; the numbers correspond to BinBase identifiers of unannotated compounds [42]. No metabolite-pairs showed opposing correlation directions between the two networks.

We next analyzed network similarity. The average degree (k), or number of connections per metabolite, was higher in Expt. A (k = 3) than Expt. B (k = 1) suggesting more genetically variable links within the metabolite network within Expt. A than in Expt. B. All measures of connectivity were significantly higher in Expt. A than in Expt. B. This included straight connectivity (P<0.001), closure of triads (P<0.001) and degree distributions (P<0.001; Figure S1). This suggests that there is a fundamental shift in the shape of the genetic networks controlling metabolism that partly explains the differences between the two experiments.

**Figure 4:**
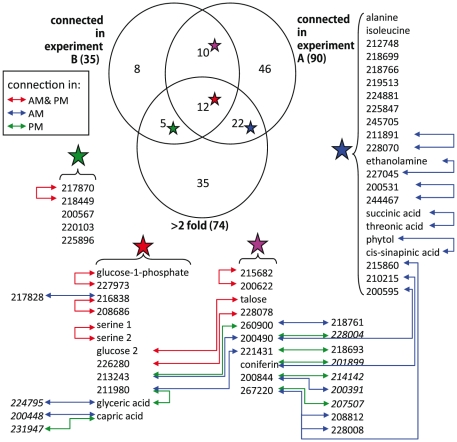
Differentially-abundant and differentially-connected metabolites. Of a total of 194 metabolites detected in both experiments, 138 were either present at more than two-fold difference (Bonferroni-adjusted P<5%) between Expt. A and Expt. B or are significantly connected (local FDR<20% using partial correlation) to at least one other metabolite in Expt. A or Expt. B. Colored stars correspond to the list of metabolites belonging to one of the four overlapping groups. Unannotated metabolites are shown by their BinBase database identifiers. Lines between metabolites indicate significant correlations present in both Expt. A and Expt. B (red), only in Expt. A (blue) or only in Expt. B (green). *Italicized* metabolites are only detected in one experiment.

To test if metabolites differing in abundance between the two experiments also participate in different genetic interactions, we investigated correlations involving metabolites that differentially accumulated in the two experiments and were significantly correlated with at least one other metabolite in both experiments. Of the 39 metabolites fulfilling these requirements only eight had identical connections within both experiments (Figure 4). For the remaining 31 metabolites, we did not detect shared connections between the two different genetic networks; either because they were connected to different compounds between Expt. A and Expt. B (4 metabolites) or they were not significantly correlated with any other detected metabolites in the alternate dataset (27 metabolites). For example, the abundance of succinic and threonic acids was 3- and 6- times higher in Expt. B than Expt. A, and while these two metabolites were significantly connected to each other in Expt. A neither demonstrated significant correlation with any other metabolites in the Expt. B data set. These results imply that metabolites that were differentially abundant between these experiments were the consequence of the metabolites being involved in different environmentally-influenced genetic networks, rather than differential regulation of the same networks.

### Metabolite abundance is heritable and complex

**Figure 5:**
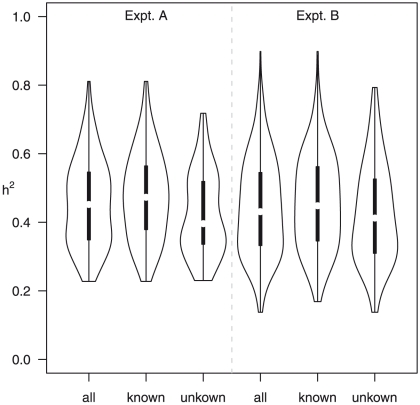
Distributions of broad-sense heritability, H^2^. Distributions are shown for Expt. A (left three violin plots) and Expt. B (right three violin plots) and for (1) all metabolites detected at the corresponding time-points, (2) known (annotated) metabolites and (3) unknown (unannotated) metabolites. The variable widths of the violin plots indicate the probability density of the data at each H^2^.

**Figure 6:**
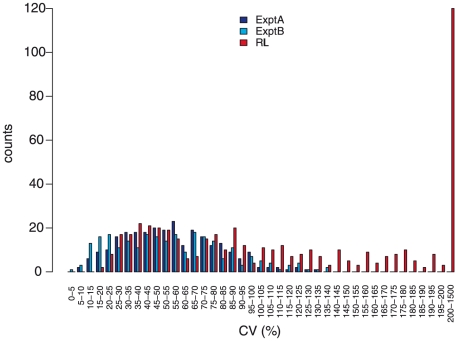
Coefficients of variation (CV) of metabolites. Shown are CV based on 96 Arabidopsis accessions in Expt. A (dark blue) and Expt. B (light blue) and across 211 Bay-0 x Shahdara RILs (red; [10]). For Expt. A & B, CV were calculated as the ratio of the standard deviation and mean across the 96 accessions.

Two lines of evidence indicate a genetic contribution to control of metabolite abundance within these two experiments and this collection of accessions. First, the estimated broad-sense heritability, H^2^, for both experiments was 0.45±0.14, with no significant difference between known and unknown compounds, suggesting our estimates are not biased by the ease of the annotation status of a metabolite (Figure 5). These estimates can be compared to heritability for traits such as yield, typically less than 10%, and flowering time, frequently greater than 90% [44]–[45]. This level of heritability provides sufficient power to detect significant genotype-phenotype associations [16], [46]–[48]. As an additional test of how genetic variation may influence metabolite variation in this collection of accessions, we estimated the genetic coefficient of variation (CV) per metabolite. Genetic CV is a dimensionless measure that allows the direct comparison of phenotypic diversity controlled by genetic variation across experiments and populations [44]. Genetic CV describes how genomic variation controls phenotypic diversity and does not reveal the contribution of individual genes to phenotype. The average genetic CV was 56%±27% for both experiments (Figure 6, Table S1).

One presumed benefit of GWAS is that the increased level of genetic variation will inherently lead to increased phenotypic variance [49]–[51]. To test this assumption we compared the genetic CV for all metabolites in the wild accessions to a structured RIL population. We obtained the genetic CV for all metabolites measured in a previously published experiment using a population of Recombinant Inbred Lines (RIL) generated from the accessions Bay-0 and Shahdara, both among the 96 accessions used in this study [10], [18]. Both RIL and GWA studies used the same GC-TOF-MS platform for data collection representing the same metabolite classes. Comparing the distributions showed that nearly one half of the metabolites had a higher genetic CV in the structured population than the collection of wild accessions;a full one third of metabolites had a genetic CV higher than the largest genetic CV present in the GWA accession collection ([10]; Figure 6). Thus, for these metabolites, a cross of two individual wild accessions provides greater phenotypic diversity than is observed in 96 wild accessions. This suggests that constraints on metabolism in wild *A. thaliana* accessions are relaxed in experimental crosses [8]. Given that there are only two accessions used for this cross, it is not surprising that some metabolites show lower genetic CV in this population than in the 96 accessions (Figure 6). For these metabolites, it is likely that a cross involving two different accessions known to differ in accumulation of these metabolites would provide a dramatic boost in genetic CV, a concept not too different to heterosis (Figure 6). This is supported by the observation that the CV per metabolic network differed between the two experiments, suggesting also that changing the environment can reveal novel genotype-phenotype associations or entire genetic networks, independent of the type of population employed for analysis (Figure S5). Thus, a collection of structured populations using different wild parents may provide a greater range of phenotypic diversity than natural populations.

**Figure 7:**
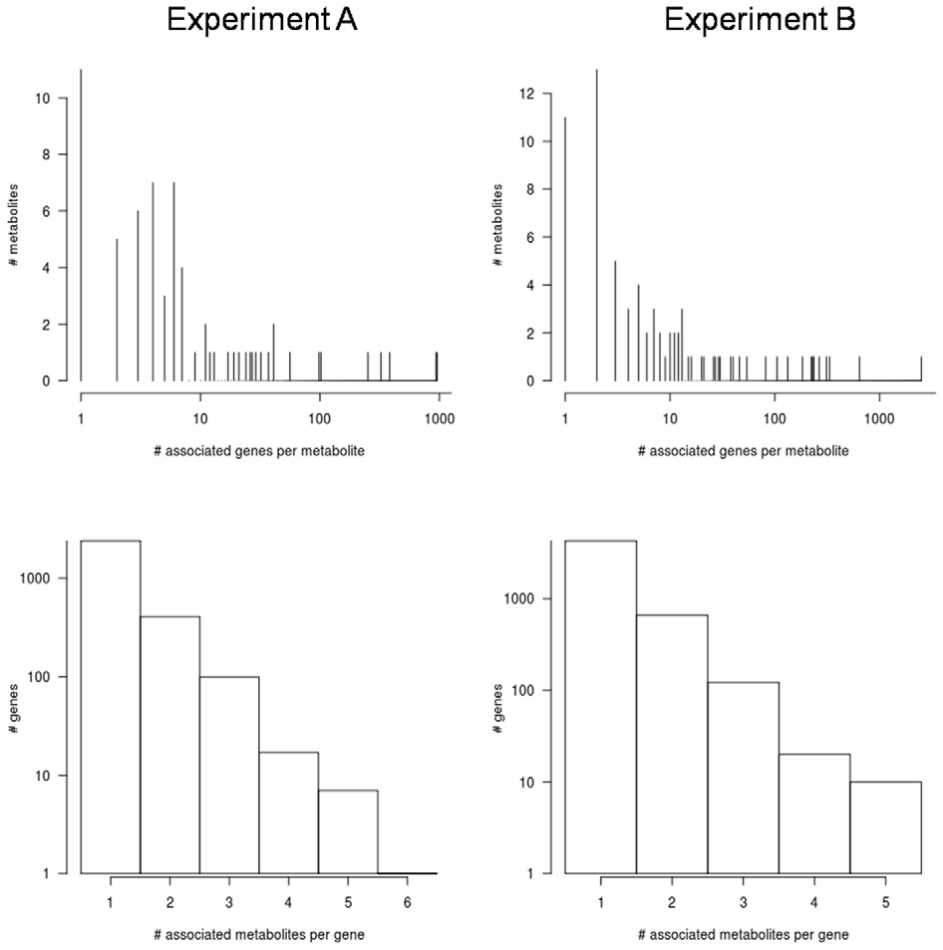
Summary of genome-wide association results. The two left panels correspond to Expt. A and the two right panels to Expt. B. Top panels show the numbers of metabolites significantly associated with the corresponding numbers of genes. The bottom panels show the numbers of genes significantly associated with the corresponding numbers of metabolites.

To further explore the genetic bases of metabolite abundance in *A. thaliana*, we searched for associations between 31,505 genes (encompassing 206,096 genotyped SNP) and the accumulation of 327 metabolites in the 96 A. *thaliana* accessions in a genome-wide association (GWA) analysis. A gene was considered to be associated with a metabolite if at least two SNP within 1 kb flanking that gene were significant at FDR<0.05. This post hoc filtering of significant SNPs on a per gene basis is based on previous observations that multiple SNPs associate with causal genes [52]. This post-hoc procedure was shown to optimize the false positive to false negative rates while correctly identifying true positive genes in a previous study of metabolic variation using these 96 accessions [17]. Less than 31% of the metabolites (67 in Expt. A, 78 in Expt. B, 18 in both) showed significant association with any gene. This was unexpected, given the relatively high (45%) heritability observed. This is also puzzling given the ability of linkage disequilibrium and population structure within *A. thaliana* to generate false-positive linkages [17]–[18]. Detection of few significant associations for traits with high heritability is consistent with the ‘infinitesimal model’ [53], suggesting that each metabolite is controlled by numerous genes with small effects. The applicability of this model is further supported by the fact that metabolites with significant associations were associated with multiple genes (A: 54±171, B: 78±297; Figure 7). While the majority of such cases are caused by local linkage disequilibrium (multiple associated genes are co-localized), there is a minority of cases where gene-gene interaction (co-associated genes are either not in LD or are in trans-LD) cannot be ruled out (see next section). Differences in metabolite abundances between the two experiments suggest few associations will be common if differential abundance is due to different genetic controls. Of the 194 compounds detected in both experiments, only one metabolite associated with the same genes in the two experiments: the unknown metabolite 244578 was associated with two tandem genes on chr5 (AT5G09310 is unannotated and AT5G09320 is a potential Rho guanyl-nucleotide exchange factor). Given the false-positive and false-negative issues with GWA, the biological effect of one or both of these two genes on metabolite 244578 requires experimental validation [17]. This single shared genetic association between the two experiments is dramatically lower than the number of shared associations expected by chance alone as estimated by permutation analyses. These GWA results and the similar heritability estimates between the two experiments argue that the *major* genetic variants controlling the detected metabolite variation among accession are likely subject to genotype x environment interactions. Genotype x environment interactions have demonstrated importance in determining plant metabolite levels [8], [27]–[29], [54]–[57].

### Metabolites are mapped to genes in local-LD and trans-LD

The numbers of significantly associated genes far exceeded the numbers of significantly associated metabolites in both experiments. In expression QTL-mapping studies (currently the most studied quantitative genomics system) the number of QTL per expression-trait generally averages less than four [58]. Indeed, a permutation analysis with our current data predicted that, on average, each metabolite would falsely associate with 3 genes in Expt. A and 5 genes in Expt. B. Instead, each metabolite in the real data demonstrated significant association with an average of 14 (Expt. A) and 24 (Expt. B) genes. Exceeding expectation, 45 metabolites were associated with more than three genes in Expt. A and 42 metabolites were associated with more than five genes in Expt. B (Figure 7). Further, six metabolites in Expt. A (asparagine, beta-alanine, and four unknown compounds) and 12 in Expt. B (ascorbic acid, threitol, trehalose, serine, coniferin, and seven unknown compounds) were associated with >100 genes. These findings strongly suggest gene-gene dependence such as local or trans-linkage disequilibrium with causal polymorphisms as previously identified for a number of *A. thaliana* phenotypes using GWAS [17]–[18]. Alternatively, epistatic interactions may create multiple associations per metabolite [10]. The absence of metabolite associations with single or few highly significant SNPs suggests that, in contrast to phenotypes such as gene-for-gene mediated disease resistance, genetic control of metabolite levels is likely complex and polygenic [18].

Of the metabolites associated with more than the expected number of genes, the majority were associated with multiple genes in close physical proximity. Of these, an average of 60% (Expt. A) and 58% (Expt. B) of the genes significantly associated with the same compound were within 10 kb of each other. These results support previous observations of association clusters within *A. thaliana* [18], [59]. Thus, it is likely that the majority of these linked associations are due to either natural selection or demographic influences, and that finer dissection of these blocks using traditional genetic approaches will be necessary to identify the true causal polymorphisms in these regions [18], [59].

Interestingly, a small percentage (1–2%) of genes associated with five (Expt. A) and seven (Expt. B) of these compounds were significantly associated with genes that were in LD with each other (r^2^>0.4) but located on different chromosomes (i.e. non-syntenic). As r^2^>0.4 is a conservative threshold (see Materials and Methods), it is likely that non-syntenic LD is more prevalent than estimated here. These non-syntenic correlated gene-pairs are non-randomly distributed within the genome (Figure S6). Most of these gene-pairs are genetically linked with the 5′ teleomeric end of chr5. This region on chr5 lacks of diversity (Figure S7), possibly due to a recent positive selective sweep [60]. It remains to be seen if the genetic, but not physical, linkage of multiple polymorphisms associated with metabolic variation with this chr5 sweep is a statistical artifact created by the relative lack of polymorphism in this region, or if the postulated sweep altered metabolic phenotypes. Such an effect could result either from a re-modulation of genetic variation within the rest of the genome to optimize the phenotypic consequence of this selective sweep, or from more direct selection on changes in metabolism occurring during the sweep.

### Association hotspots are in trans-LD

While we observed significant association between one metabolite and many genes, we also identified instances where multiple metabolites associated with the same genes. Permutation analyses predicted that no more than three metabolites should associate with the same gene. Yet, 25 (Expt. A) and 30 (Expt. B) genes were significantly associated with four to six measured compounds. The numbers of metabolites associated with each gene in the real datasets significantly differed from the permutation-derived null distributions, confirming the presence of association ‘hotspots’. Other ‘hotspots’ have also been observed in quantitative genomics studies of metabolite levels using structured mapping populations (e.g. RILs) [8], [10], [61].

**Figure 8:**
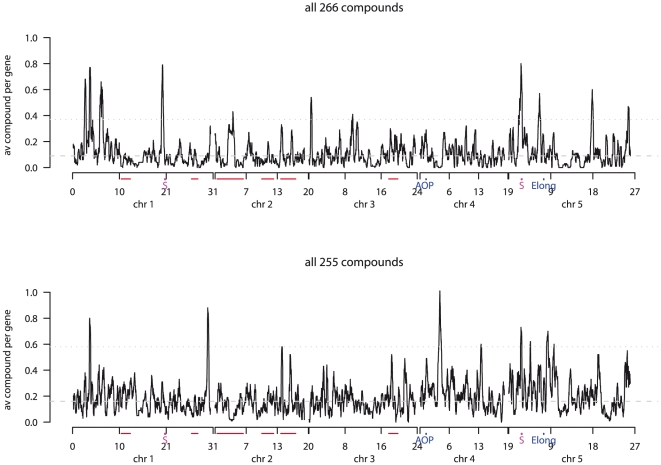
Genome-wide association hotspots. Plotted are the average numbers of associated metabolites per gene, estimated at sliding windows of 100 genes, for Expt. A (top) and Expt. B (bottom). Indicated on the axes are previously reported linkage hotspots (red lines; [10]), and two previously reported recent positive selective sweeps (pink S; [60]).

To better identify association hotspots within our GWA experiments, we estimated the average number of associated compounds per gene within sliding windows of 100 genes. Given the observed extent of LD decay within *A. thaliana*, we expected to detect associations between metabolite levels and gene clusters near the actual causal polymorphism [59]. A sliding average will accentuate regions likely to contain at least one causal polymorphism (Figure 8). Using this approach we identified 20 and 11 significant hotspots containing 129 and 60 metabolite-associated genes in the two experiments (Table S2). In Expt. A, 13 hotspots were detected on chr1, two on chr3, and five on chr5 all ranging from 1.5 to 5 kb. In Expt. B, six hotspots were detected on chr1, two on chr4, and three on chr5. Interestingly, only two hotspots, one located on chr1 containing 13 genes, were detected in both experiments, again confirming that the experiments identify different genetic contributions to metabolite variation. The second hotspot was a region previously identified as a selective sweep on chr 5 which might be expected to generate false-positive associations due to decreased diversity (Figure 8; [60]). However, as the hotspot significance threshold was obtained by conducting a permutation analysis utilizing the same SNP data but shuffling the phenotypes, this would account for this potential bias and supports the selective sweep being a hotspot for genetic variation associated with metabolic phenotypes [62]. Additionally, this region is not a hotspot for all measured phenotypes, again supporting its identification with metabolite variation in this dataset [17]–[18]. However, it remains to be tested if natural variation for any of these genes within this region control plant metabolism.

**Figure 9:**
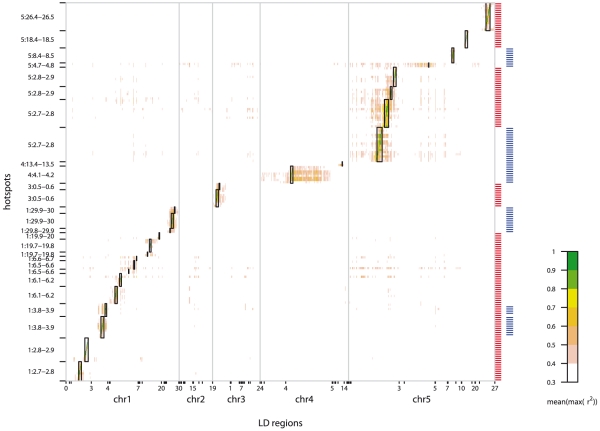
Heatmap of gene–gene LD for all hotspot genes. The average maximum SNP-SNP r^2^ (see Materials and Methods) between each of the 176 hotspot genes (y-axis) and the genome (x-axis) is plotted. Not all 31,505 genes are shown on the x-axis: only those with average maximum SNP-SNP r^2^>0.3 are included. Note that, because only selected genes are plotted, the genomic distances in the two axes are not to scale. The horizontal dashes on the right-hand side of the heatmap indicate whether the gene is located within a hotspot identified in Expt. A (red) or Expt. B (blue).

We examined the extent of LD of all gene-pairs within all hotspot regions to look for co-segregation of hotspots. The results showed that a previously proposed recent selective sweep on chr5 [60] is in strong LD with many of the metabolite hotspots (Figure 9). This is particularly true for a 2.7–2.9 Mb region which is not only in LD with many other genomic regions but also contains extended local LD. Similarly, chr1 (6.1–6.8Mbp) also appears to have elevated LD with various regions on chr2, chr3 and ch5 but has no strong local LD. The chr4 hotspots were not in LD with non-syntenic regions, but did contain strong local LD, particularly at 4.1–4.2 Mbp. Interestingly, while the chr5 hotspots in Expt. A coincided with the region of the proposed recent positive selective sweep [60], the immediately adjacent hotspot in Expt. B is not within this sweep region. Thus, it is possible that this predicted selective event has affected a gene which contributes to the differential regulation of metabolites between the two experimental conditions.

### Similar/different genetics of taxonomically similar metabolites and metabolites of the same pathway

To better understand the naturally variable genetic control of the metabolome we focused our analyses on annotated metabolites, including those that have been assigned into a metabolite (taxonomy) class (based on the Human Metabolome Database) or assigned into a known KEGG (Kyoto Encyclopedia of Genes and Genomes) pathway of *A. thaliana*. Specifically, we searched for metabolite classes and metabolic pathways that may be over- or under- represented. Considering metabolite classes, we found significantly more amino acids (11/20 with P(χ^2^)<0.03; 6 expected) and hydroxyl acids (4/5 with P(χ^2^)<0.04; <2 expected) associated with at least one gene in Expt. B but not Expt. A. No other classes of metabolites have significantly more or less than the expected numbers of compounds associated with any genes. We also found that fewer genes were shared between amino acids in Expt. A (P(χ^2^)<0.001; Figure S8) and between carbohydrates in Expt. B (P(χ^2^)<0.02; Figure S9). Compounds significantly associated with the same gene (gene-sharing) did not belong to any particular metabolite classes. We infer from these results that 1) different genetic effectors control metabolite abundance under the two experimental conditions, 2) amino acids and carbohydrates may have more coordinated genetic control in one experimental condition than another, and/or 3) individual amino acids and carbohydrates, under these two conditions respectively, are likely to be governed by different genetic determinants; i.e. individual compounds within these classes may share few genetic regulators.

Considering biochemical pathways, six pathways identified two or more compounds associated with the same polymorphic genes. These included galactose metabolism, ascorbate metabolism and aspartate metabolism, each having 2–3 of their members significantly associated with an average of three genes. The function of many of these genes is not currently known, but with continuing improvements in genome annotation, the use of GWA to analyze biochemical networks will increase precision and/or power to detecting causal polymorphisms.

## Discussion

Although the genetic architecture of individual and small targeted classes of metabolites has been well-studied [4], [12], [56], [59], [63]–[65], the genetic architecture underlying natural variation in the metabolome as a whole remains poorly understood. Recent studies have explored the genetic architecture of the metabolome [8], [10], [61], but this has not extended to genome-wide association studies. In this paper we described natural variation of the metabolite profiles of 96 accessions of *A. thaliana* in terms of abundance, genetic correlation, and genetic association. Genetic variation is a major component controlling the *A. thaliana* metabolome. We generated two metabolomics datasets, which differed essentially only at the time of tissue harvest, to explore the extent of environmental effects and its interactions with genetics on metabolite abundance.

Clear differences between these two datasets were observed, suggesting large differences in the metabolic dynamic related to environmental conditions. While attempts were made to keep all non-experimental variables constant, we recognize the possibility of other environmental differences between these two experiments. As such, we refrain from drawing the conclusion that the differences in metabolic profiles were due solely to the time of harvest. Without loss of generality, we conclude instead that the genetic network controlling *A. thaliana* metabolism is likely to be conditional upon the environment as well as parameters such as tissue and plant age [28], [30]. Thus, future experiments should include factorial analysis of environmental perturbations to better understand links between genotype and phenotype.

### Association mapping versus structured populations

Compared to previous QTL-mapping studies using approximately 200 RILs, where 40%–75% of all examined metabolites were mapped to at least one QTL [10], [61], the current GWA study, using 96 accessions, found significant association of only 23%–30% of all detected metabolites with at least one region of the genome. A number of factors might explain this discrepancy. First, GWA and traditional QTL studies usually differ in statistical power. It is generally proposed that fewer individuals are necessary when using natural compared to structured populations due to the increased potential for recombination to isolate causal polymorphism in the former, as lineages are separated by many generations [66]. However, it is unclear to what extent the increase in recombination opportunity among our 96 accessions compensates for the increased statistical power provided by a structured population containing twice as many genotypes. Secondly, while a greater number of recombination events should facilitate fine mapping of a trait, the reduction in linkage disequilibrium between the causative polymorphism and its closest marker will weaken the phenotype-genotype (trait-marker) association, especially if the effect is small. As such, elevated genetic diversity in the GWA population may actually decrease our statistical power to associate that diversity with trait variation [67]–[68] if the observed range of metabolite phenotypic diversity within the GWA population is lower than in the RIL, as we observed here (Figure 5). Thirdly, *A. thaliana* RILs have shown a high level of epistasis controlling metabolic traits, which may decrease our ability to identify significant associations in GWA [10]. While different metabolite detection technologies (e.g. LC-MS vs. GC-TOF-MS) can bias the identified classes of metabolites (e.g. primary vs. secondary) and differing selective forces are suggested to shape variation in primary versus secondary metabolite levels, both the RILs and GWA were analyzed using GC-TOF-MS [4], [10], [61], [69]. A combination of GWA and QTL-mapping studies are likely necessary to fully query the genetic architecture of the metabolome.

### LD and causal gene identification

We identified numerous metabolites that significantly associated with hundreds of genes. These multiple associations may be real, as could result from epistatic genetic controls, or spurious, as would result from local linkage disequilibrium. For the majority of these associated genes, we show that a significant fraction of gene associations with metabolite traits likely results from local linkage disequilibrium with a causal polymorphism. Previous work with secondary metabolites showed that GWA hotspots, regions associated with multiple metabolic traits, are likely not spurious, but instead contain at least one causal gene [59]. As such, while local LD prevents direct identification of causal gene(s), it provides strong support for the presence of at least one causal polymorphism within these regions. Future work will be required to identify ways to distinguish causal from non-causal polymorphisms.

In addition to local LD we also showed that a portion of genes associated with the same metabolite are actually located on different chromosomes; i.e. in trans-LD. Interestingly, previous work with secondary metabolites has shown that trans-LD between genes may predict epistatic relationships between gene pairs [59]. Given the prevalence of epistasis in *A. thaliana* metabolism [4], [10], [59], it is possible that genes showing trans-LD and significant associations with the same metabolite interact to determine the level of the metabolite. Detection of multiple genes that are in local LD and concurrently associated with the same trait is a well documented phenomenon in QTL-mapping, however future experimental efforts to validate candidate gene-trait associations will be required to explore the intriguing question of why some metabolites are associated with genes in trans-LD. Response of physically distant regions to a selective sweep (in effect, hitch-hiking of distant loci), as postulated for groups of genotype–metabolite associations in non-syntenic LD with a previously-identified selection sweep on chr5, is a phenomenon requiring validation and thorough exploration in additional systems, as it would potentially complicate interpretation of both evolutionary and quantitative genetic analyses.

### Genetic network structure

Interestingly, while most previously described metabolic networks have shown exclusively scale-free properties [24], [70], our metabolic-networks, reconstructed using Spearman's correlation, suggested a network topology somewhere between random and scale-free (Figure S1). The discrepancy between our findings here and previous studies may be explained by the difference in objects of correlation analyses: previous studies correlated metabolite abundance across replicate measures, whereas this study estimated genetic correlations across *A. thaliana* accessions. Additionally, previous analyses of metabolic-networks have queried laboratory-generated mutations in environments with reduced selection whereas our networks were obtained using natural polymorphisms likely to be shaped by natural selection. As such, it is possible that the metabolome is in fact a single unique network but that natural selection only allows natural polymorphism to persist within a portion of this network; it is only this variation that is detectable. Again, this creates a major difference in the interpretation of the estimated networks. Even the weak scale-free network properties disappeared when a partial correlation approach was adopted, suggesting a much smaller proportion of the metabolites measured actually share direct regulators that are genetically diverse, and have regulatory effects strong enough to be detectable with the experimental power in this study.

### Future directions

Metabolomics and quantitative genetics show great potential to help better link genetic variation with phenotypic variation. The observations described here suggest that the use of GWA to identify these links is highly susceptible to genotype x environment effects, as well as epistatic interactions and their potential effects on population structure. As such, populations may need to be phenotyped in a broad set of environments to fully query genetic control of the observed natural variation in phenotypes. This dataset encourages further investigation of the environmental sensitivity of the basic genetic network architecture. The observation of GWA hotspots, while confounding our ability to directly find causal genes, suggests an approach to find regions that will contain a causal gene. Further work is required to test the hundreds of candidate genes identified with this approach to see if there are ways to rank candidate genes within these hotspots.

## Materials and Methods

### Growth conditions and plant material

A collection of 96 previously described *A. thaliana* accessions was examined. Seeds were imbibed and cold stratified at 4°C for three days to break dormancy. Four plants of each accession were grown in individual pots in a randomized block design. The full experiment was replicated over two years utilizing the same growth chamber providing four metabolomics assays per accession per replicate. For all experiments, plants were grown in flats with 36 cells per flat, and maintained under short day conditions in controlled environment growth chambers. At 35 days post germination, a fully-expanded mature leaf was harvested, digitally photographed and metabolite extraction and profiling were performed as described below [10]. In the first replicate (Expt. A), all harvesting started at subjective mid-day, finishing within two hours, and in the second replicate (Expt. B), all harvesting started two hours prior to subjective night-fall, finishing within two hours. In all, there is at least a six hour difference in the harvest time between the two experiments. Given the time between experiments, the batch of soil necessarily changed and the experiments, while performed in the same environmental chamber, were at different times of year. Each plant was independently harvested, in random order to minimize any variation due to harvest order, and extracted as per published protocols providing a total of 768 samples, four per 96 accessions per two experiments [2], [24], [42], [71]. Tissue samples were stored dry at −80°C until automated derivatization and GC-TOF-MS analysis at the UC Davis Genome Center Metabolomics Facility (http://metabolomics-core.ucdavis.edu; [42]).

### Metabolite profiling

For each metabolomics sample, one leaf disk from each of two leaves per plant was harvested, providing two leaf disks of approximately 20 mg total weight. Metabolite identity was determined by comparing retention time and mass to the 2007 UC Davis Genome Center Metabolomics Facility metabolites database (http://fiehnlab.ucdavis.edu/Metabolite-Library-2007; [42]). At the time of analysis, this library contained reference spectra for 713 known metabolites, generated by the analysis of purified reference compounds. The ion count values were used as a surrogate for metabolite abundance.

### Metabolomics data pre-processing

Following the initial quality control which detected 416 compounds in at least 50% of the samples in at least one accession across both experiments, the data was further processed, independently for the two experiments, to include 1) only compounds present in >50% of the samples per accession, and 2) only samples where at least 50% of the predominant compounds (compounds detected in at least 70% of the samples) were detected. This resulted in 266 compounds in 326 samples (Expt. A) and 255 compounds in 282 samples (Expt. B).

Due to quasi-gamma distributions of some metabolites, all ion count values were log_2_-transformed. To minimize potential daily variation in MS sensitivity, median-normalization (and standardized to 9 log_2_ units) was performed across the date of the GC-TOF-MS runs: eight runs for Expt. A and 15 runs for Expt. B.

### Estimation of heritability, genetic means, and genetic coefficient of variation

Broad-sense heritability (H^2^) was estimated as the variation attributable to accession (A) variations whilst accounting for variations due to population structure (S) and experimental noise (F for plotting flat & R for replication). Specifically, H^2^ was estimated as the A_a_(S_s_) type II sum of squares per the linear model: y_safr_ ∼µ+S_s_+F_f_+A_a_(S_s_) +R_r_(F_f_) +ε, where y_safr_ is the log_2_-value of the metabolite corresponding to the sample belonging to accession A_a_ (a = 1,..,96) and population structure S_s_ (s = 1,..,8), that was planted in flat F_f_ (f = 1,2) as replicate R_r_ (r = 1,..,4). Residual error was assumed normally distributed: ε ∼ N(0, σ_ε_^2^). The model was run independently for each dataset.

Accession means (average value per accession per trait) were estimated using a similar linear model with the exclusion of S_s_: y_afr_ ∼ µ+F_f_+A_a_+R_r_(F_f_) +ε. The coefficients of the A_a_ terms were taken as the genetic means. We do not account for population structure effect here because it will be inherently accounted for in the association mapping analysis (below). These accession means were used for all subsequent analyses (Datasets S1 and S2).

The accession means as well as previously published means for a single RIL population were also used to estimate genetic coefficient of variations (CV) as a way of comparing the level of genetically determined phenotypic diversity between the different populations. The genetic CV was independently calculated for each metabolite by taking the standard deviation across the genotypes and dividing it by the mean; σ/µ [69], [72]–[73].

### Metabolite correlation analysis

Zero-order correlation between all metabolite-pairs was estimated using Spearman's Correlation Coefficient. Significance was determined using Student's t-distribution: t = ρ/√( (1-ρ)/(n-2) ) and local (density-based) false discovery rate was estimated using the R/fdrtool package [74]–[75].

First-order correlation was estimated using a Static Shrinkage approach of Partial Correlation [76] via the R/GeneNet package [36]. Local false discovery rates were estimated using the network.test.edges function of the same package.

Metabolite networks from correlation matrices were generated using the R/igraph package [77]. The same package was also used to calculate the various network properties, including degree (k) and clustering coefficient (C(k)).

### Genome-wide association mapping

Genotypes of ∼250,000 SNP of each of the 96 accessions were obtained from the Arabidopsis 2010 Project (http://walnut.usc.edu/2010/data; [50], [78]). Single-locus GWA mapping was performed using a mixed-model approach, EMMA [79], where the effect of each SNP on a metabolite was modeled as a fixed-effect. The effect of population structure was also included in this model as a random-effect and is represented as a genetic similarity matrix (estimated using the SNP genotypes). Variance-components to this model were estimated directly using maximum likelihood as implemented in the R/EMMA package [79]. For this analysis, accession means were used and performed independently for the two experiments. P-values for all SNP-by-metabolite tests were extracted from EMMA. P-value distributions were roughly uniform (data not shown). Q-values were estimated using R/qvalue [80]–[81]. Significant SNP-metabolite association was defined at q<0.20.

Based on the genomic location of each SNP we identified a subset 206,096 SNP, with greater than 5% minor allele frequency in this collection of 96 accessions, residing within 1 kb of at least one of 31,505 genes. The statistical significance of each SNP was independently calculated, followed by post-hoc filtering to identify the top candidate genes linked with specific metabolites. A gene-metabolite link was called a candidate if ≥2 SNP within 1 kb of the gene were significantly associated with the metabolite. This protocol was previously described using a list of known causal genes controlling differential abundance of metabolites within these 96 accessions [17] and relies upon previous observations that multiple SNPs per causal gene show statistical association with a phenotype [52]. The use of ≥2 SNP within 1 kb of the gene was found to optimize the ratio of false negative and false positive results while maintaining the maximal number of true positive candidates [17].

Association hotspots were determined as follows: 1) the number of significantly associated metabolites was counted for each gene, 2) sliding averages of these counts at 100 gene-intervals were calculated, 3) the genes with sliding averages exceeding the estimated maximum average number of false compounds per gene were considered as hotspot genes (448 for Expt. A and 239 for Expt. B), 4) genes whose immediate neighbor *not* passing the same threshold were excluded, leaving 381 genes in 101 intervals (Expt. A) and 215 genes in 56 intervals (Expt. B), 5) only hotspot intervals with a) an average of more than two compounds per gene or b) at least eight genes within the interval with an average of more than one compound per gene were maintained. This resulted in a final set of 20 hotspots (129 genes) in Expt. A and 11 hotspots (60 genes) in Expt. B (Table S2).

Permutation datasets for each experiment were generated for assessing false associations. Permutation was performed for the purpose of breaking true gene-metabolite associations, and so accessions were re-sampled without replacement within each compound. This is similar to permutation for QTL mapping wherein the phenotypes are shuffled randomly across the defined genotypes [62]. For each permutation, GWA mapping as described above and similar subsequent analyses as performed for the real data were performed independently. Permutations were conducted independently for Experiment A and B. The “maximum average number of false compounds per gene” was estimated from this permutation analysis: the numbers of significantly associated metabolites per gene were counted, and the sliding averages at 100-gene-intervals were also calculated and the maximum of these sliding averages was the “maximum average number of false compounds per gene”.

### Estimation of linkage disequilibrium

Linkage disequilibrium was estimated for all pairs of 6,783 significantly associated genes. Due to heavy computation demand, each gene was collapsed into a biallelic locus prior to LD calculation: for each gene, all empirically observed haplotypes were first deduced from its SNP genotypes then the haplotype with the highest frequency was assigned as the “major” allele and all other haplotypes as the “minor” allele. Because of the homozygous nature of the accessions, this resulted in each accession having a homozygous biallelic genotype at each gene. This new genotype matrix was then used to calculate the r^2^ LD measure. r^2^>0.4 was used as a very conservative arbitrary threshold: as a comparison the 99^th^ percentile of all non-syntenic (pairs of genes *not* on the same chromosome) LD was 0.12 and the largest non-syntenic LD was 0.62; only 74 non-syntenic gene-pairs exceed this threshold.

LD between each of the 176 hotspot genes and 31,505 genes in the genome were calculated as the average maximum SNP-SNP r^2^. All SNP within 1 kb of each gene were first identified: 1,624 unique SNP within at least one hotspot gene and 206,096 unique SNP within at least one of all genes. For each of 176×31505 = 5544880 gene-pairs, 1) r^2^ were calculated between all corresponding SNP-pairs, 2) maximum r^2^ at each SNP of a gene was determined separately for each of the two genes, and 3) the two maximum values were averaged to give the gene-gene r^2^ value. A gene-pair is defined as in significant LD if their corresponding r^2^ exceeded the average non-syntenic r^2^ = 0.323, calculated from all gene-pairs located on *different* chromosomes. The gene-gene r^2^ calculations will be somewhat elevated by using the maximum value per SNP but then restored to a slightly conservative estimate by averaging across SNPs. This should be less influenced by individual pairwise LD values, as a maximal r^2^ is only 1.0 and the genomic average between any two SNPs is about 0.20–0.33. Additionally, examining blocks of genes effectively hides extreme individual gene values, diminishing the effect of individual outliers. While this is meant to represent the potential trans-LD between these regions, intensively computational matrix approaches are required to test if these are truly biological outliers.

### Metabolite classes and pathways

Metabolite classes considered in this paper were defined according to the Human Metabolome Database (http://www.hmdb.ca/public/downloads/current/metabocards.zip). Metabolites were also assigned to zero or more *A. thaliana* specific pathways according to KEGG (ftp://ftp.genome.jp/pub/kegg/pathway/organisms/ath/ath.list and ftp://ftp.genome.jp/pub/kegg/pathway/map_title.tab).

### Other test statistics

Differential metabolite abundance was tested using Two Sample Paired t-test for each metabolite detected in both trials. Over-representation of metabolite in specific classes or pathways was assessed using Pearson's χ^2^ test for all compounds assigned to a metabolite class or at least one metabolic pathway.

## Supporting Information

Dataset S1 Table of adjusted accession means of 266 metabolite compounds for the 96 accessions in experiment A.
(0.44 MB TXT)

Dataset S2 Table of adjusted accession means of 255 metabolite compounds for the 96 accessions in experiment B.
(0.42 MB TXT)

Figure S1 Metabolic-network properties for experiment A (left panels) and experiment B (right panels). Networks were generated from all significant metabolite-metabolite correlations at local FDR <5% using Spearman's ρ correlation. Top panels show the relationship between clustering coefficient, C(k), and degree, k. Degree is the number of connections per metabolite (node). C(k) is the proportion of all triplets (simultaneous connection to two other metabolites) that are closed (all three metabolites are connected to each other). Bottom panels show the degree distributions, P(k); the insets show the same relationship at a log-log scale.
(0.26 MB TIF)

Figure S2 Connectivity between experiments. Average connectivity per node (average number of connected metabolites per metabolite) corresponding to r*2* cut-offs of 0.35 - 0.95 is shown for experiment A (black) and experiment B (red).
(0.17 MB TIF)

Figure S3 Genetic correlations of metabolite-pairs. For comparison, only the 194 metabolites detected in both experiment A (left) and experiment B (right) are shown. Metabolites are order identically for datasets, and only annotated metabolites are labeled. Colors denote Spearman's ρ correlation coefficients ranging from negative correlations, -1, (red) to positive, +1, (blue).
(0.58 MB TIF)

Figure S4 Distributions of correlation coefficients. The distributions of all metabolite-metabolite correlations as determined by Spearman's ρ (solid lines) and partial correlation coefficient, r (dashed lines), are shown for experiment A (back) and experiment B (blue).
(0.16 MB TIF)

Figure S5 Distributions of coefficients of variations (CV) of subsets of metabolites across 96 Arabidopsis accessions in the AM (left) and PM (right) experiments. Compared are all detected metabolites (black), metabolites that are differentially abundant between experiments (red), carbohydrates (green), metabolites involved in galactose metabolism, KEGG ID: ath00052 (blue), metabolites involved in the urea cycle, KEGG ID: ath00220 (cyan), and metabolites involved in starch and sucrose metabolism, KEGG ID: ath00500 (pink).
(0.26 MB TIF)

Figure S6 Non-syntenic LD between genes concurrently associated with the same metabolite for the A (left) and B (right) experiments. Shown are gene-pairs (arbitrarily assigned as gene 1 and gene 2) that are (i) associated with the same metabolite, (ii) located on different chromosomes, and (iii) are in strong LD (r^2^>0.4). Genes 1 and 2 are physically ordered along the two parallel lines. The same colored lines connected the gene-pairs indicate that the genes were associated with the same metabolite.
(0.52 MB TIF)

Figure S7 LD between the Chr5 recent positive selective sweep and the genome. r^2^ was calculated for all 219 SNP within the sweep region of Chr5:2,790,000-2,900,000 against all of ∼ 250,000 available SNP across the genome. The sliding averages of the medians (black) and the 25th and 75th percentile (grey lines) in 50 SNP-intervals are plotted.
(0.05 MB TIF)

Figure S8 Significant gene association rate with amino acids. Proportion of the genes showing significant association with the corresponding numbers (x-axis) of metabolites for all 266 metabolites (white) and for 20 amino acids (hashed bars).
(0.05 MB TIF)

Figure S9 Significant gene association rate to carbohydrates. Proportions of genes significantly associated with the corresponding numbers (x-axis) of metabolites for all 255 metabolites (white bars) and for 15 carbohydrates (shaded bars).
(0.05 MB TIF)

Table S1 Information and summary statistics of metabolites. A tab-delimited file of a 14-column table containing: BinBase identifier of each metabolite (ID), the metabolite name (Name), Retention Index and quantitative mass (quantmass) from the GC-TOF-MS, KEGG identifier (KEGG), the Human Metabolome Database metabolite taxonomy classification (Taxonomy Class), the average abundance in the A trial (A Mean), the average abundance in the B sample (B Mean), the paired t-test statistic, the degrees of freedom of the test-statistic (d.f.), the corresponding P-values, the Fold Difference, the coefficients of variation (CV) in the A and B data.
(0.44 MB TXT)

Table S2 Lists of the 129 genes residing within 20 association hotspots detected in experiment A and of the 60 genes residing within 11 association hotspots detected in experiment B. This is a tab-delimited text file where each row corresponds to a metabolite and the columns correspond to: the experiment (A or B), a Hotspot identifier within which the gene is contained, the gene's TAIR identifier, Gene symbol, chromosome of the gene, the gene's starting genomic position, the gene's ending genomic position, the number of significantly associated SNP (NumMetabs), the number of SNP within 1kb of the gene (NumSNP_within1kb), and the average minor allele frequency of the SNP within 1kb of the gene (MeanMAF_within1kb).
(0.42 MB TXT)
