# Supplementary figures and images for: The Complex Genetic Architecture of the Metabolome
Source: PLoS Genet. 2010 Nov 4;6(11):e1001198. doi: 10.1371/journal.pgen.1001198 (PMC2973833; doi:10.1371/journal.pgen.1001198)

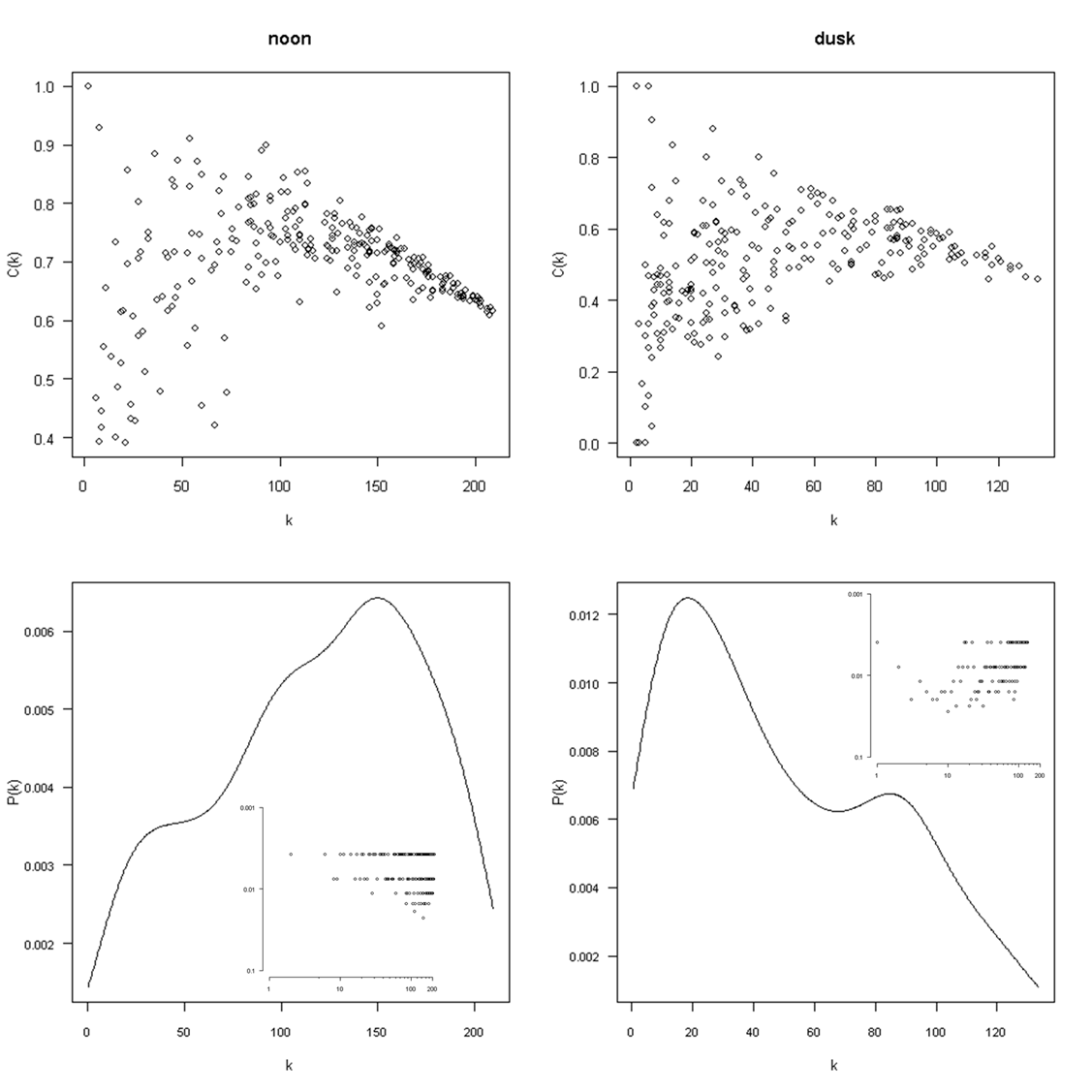

Supplement: Figure S1 — Metabolic-network properties for experiment A (left panels) and experiment B (right panels). Networks were generated from all significant metabolite-metabolite correlations at local FDR <5% using Spearman's ρ correlation. Top panels show the relationship between clustering coefficient, C(k), and degree, k. Degree is the number of connections per metabolite (node). C(k) is the proportion of all triplets (simultaneous connection to two other metabolites) that are closed (all three metabolites are connected to each other). Bottom panels show the degree distributions, P(k); the insets show the same relationship at a log-log scale. (0.26 MB TIF) [file pgen.1001198.s003.tif]

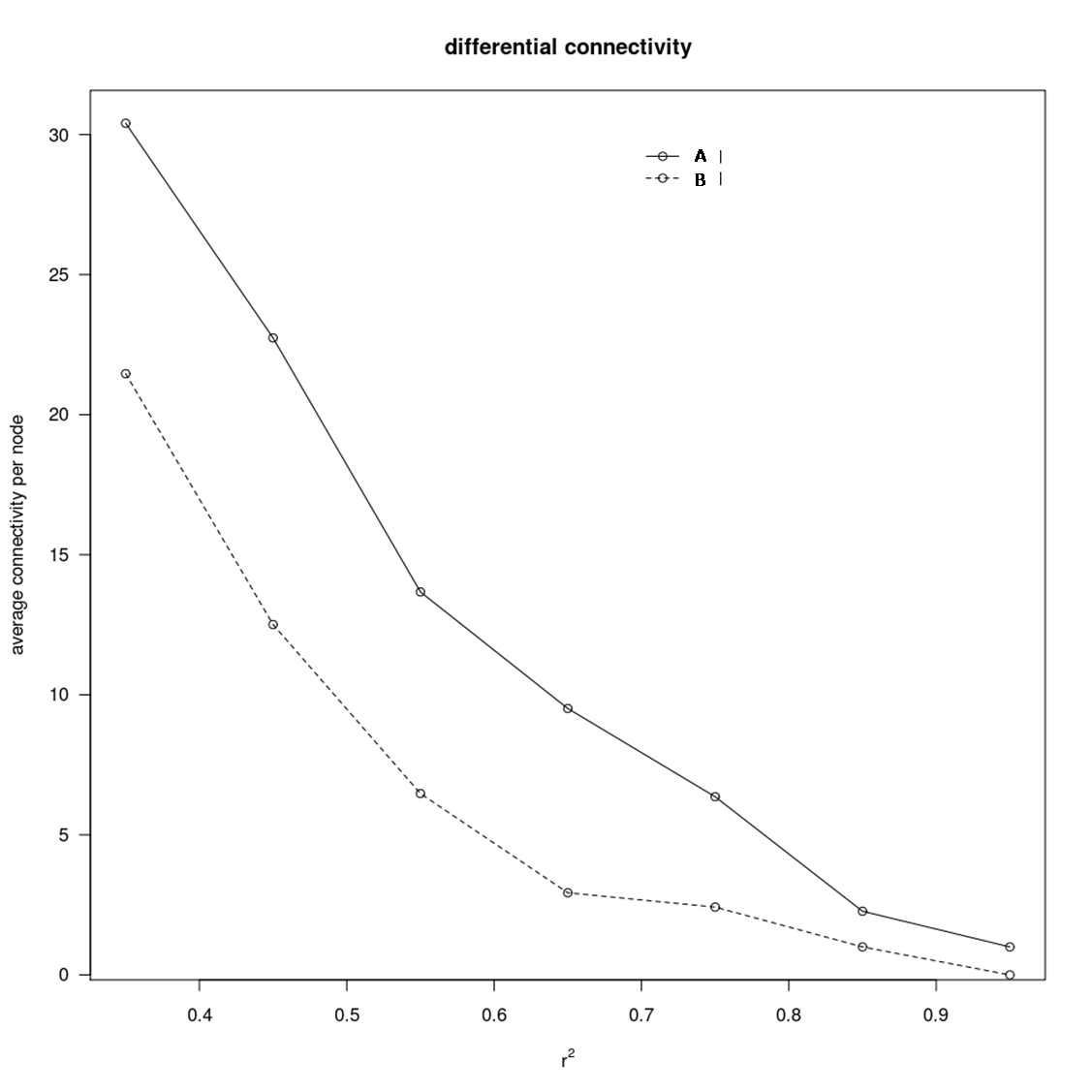

Supplement: Figure S2 — Connectivity between experiments. Average connectivity per node (average number of connected metabolites per metabolite) corresponding to r2 cut-offs of 0.35 - 0.95 is shown for experiment A (black) and experiment B (red). (0.17 MB TIF) [file pgen.1001198.s004.tif]

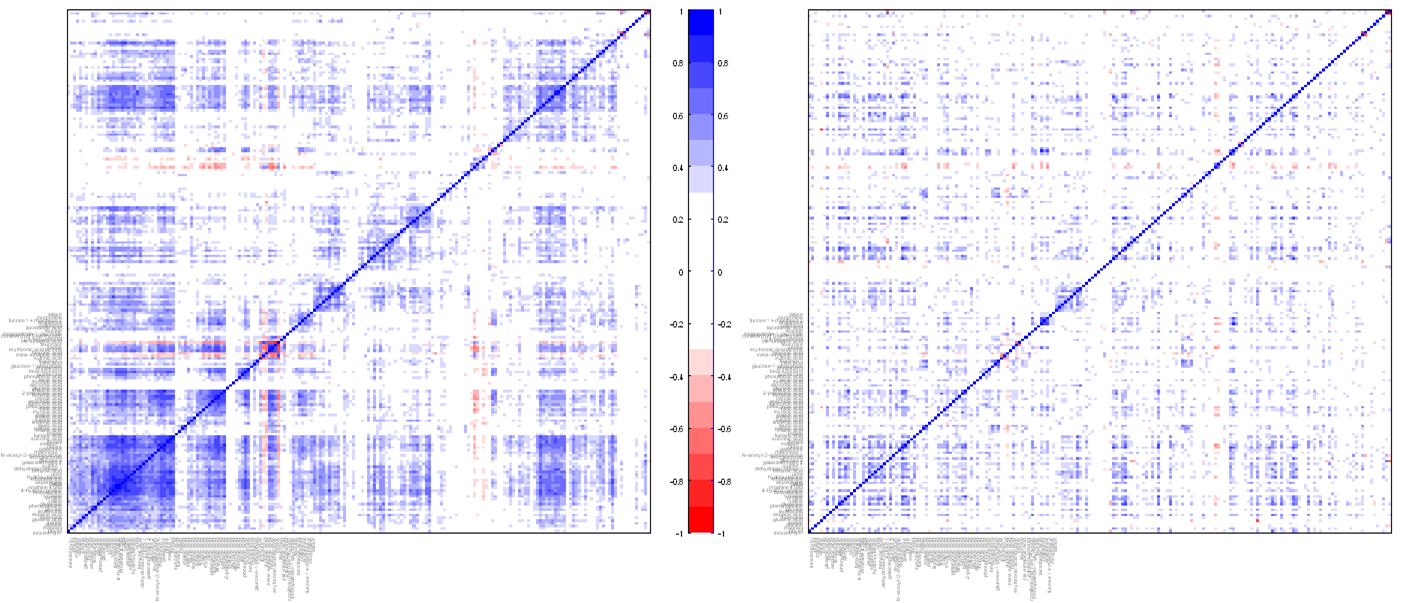

Supplement: Figure S3 — Genetic correlations of metabolite-pairs. For comparison, only the 194 metabolites detected in both experiment A (left) and experiment B (right) are shown. Metabolites are order identically for datasets, and only annotated metabolites are labeled. Colors denote Spearman's ρ correlation coefficients ranging from negative correlations, -1, (red) to positive, +1, (blue). (0.58 MB TIF) [file pgen.1001198.s005.tif]

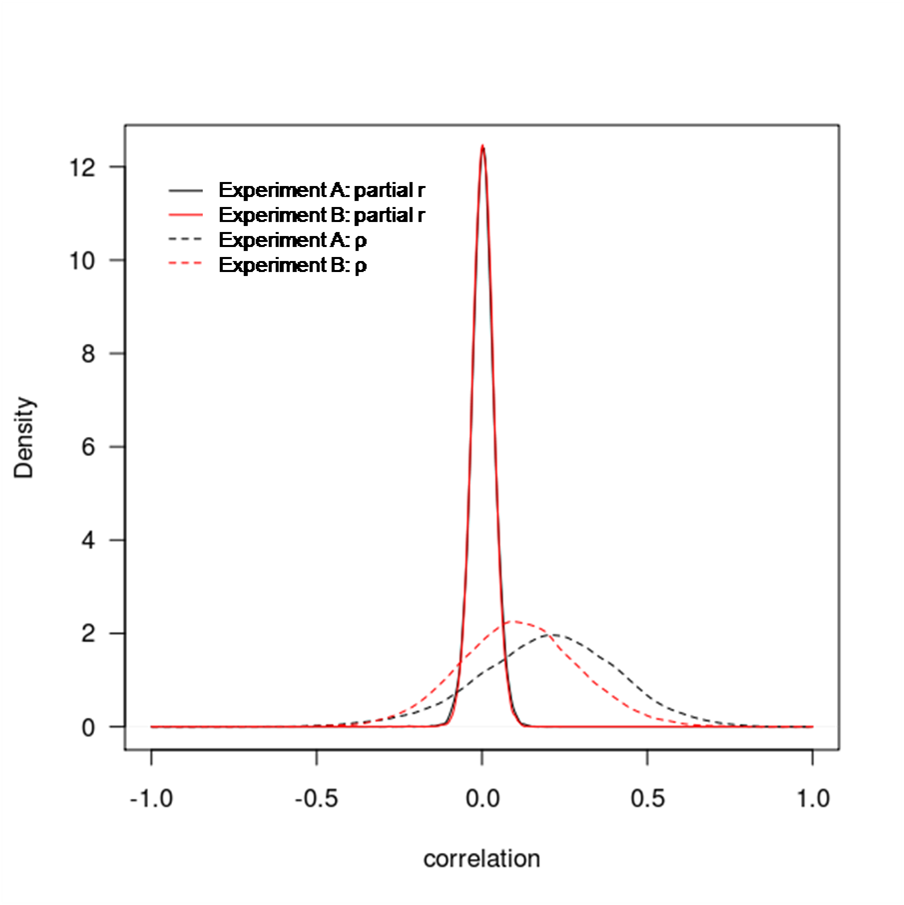

Supplement: Figure S4 — Distributions of correlation coefficients. The distributions of all metabolite-metabolite correlations as determined by Spearman's ρ (solid lines) and partial correlation coefficient, r (dashed lines), are shown for experiment A (back) and experiment B (blue). (0.16 MB TIF) [file pgen.1001198.s006.tif]

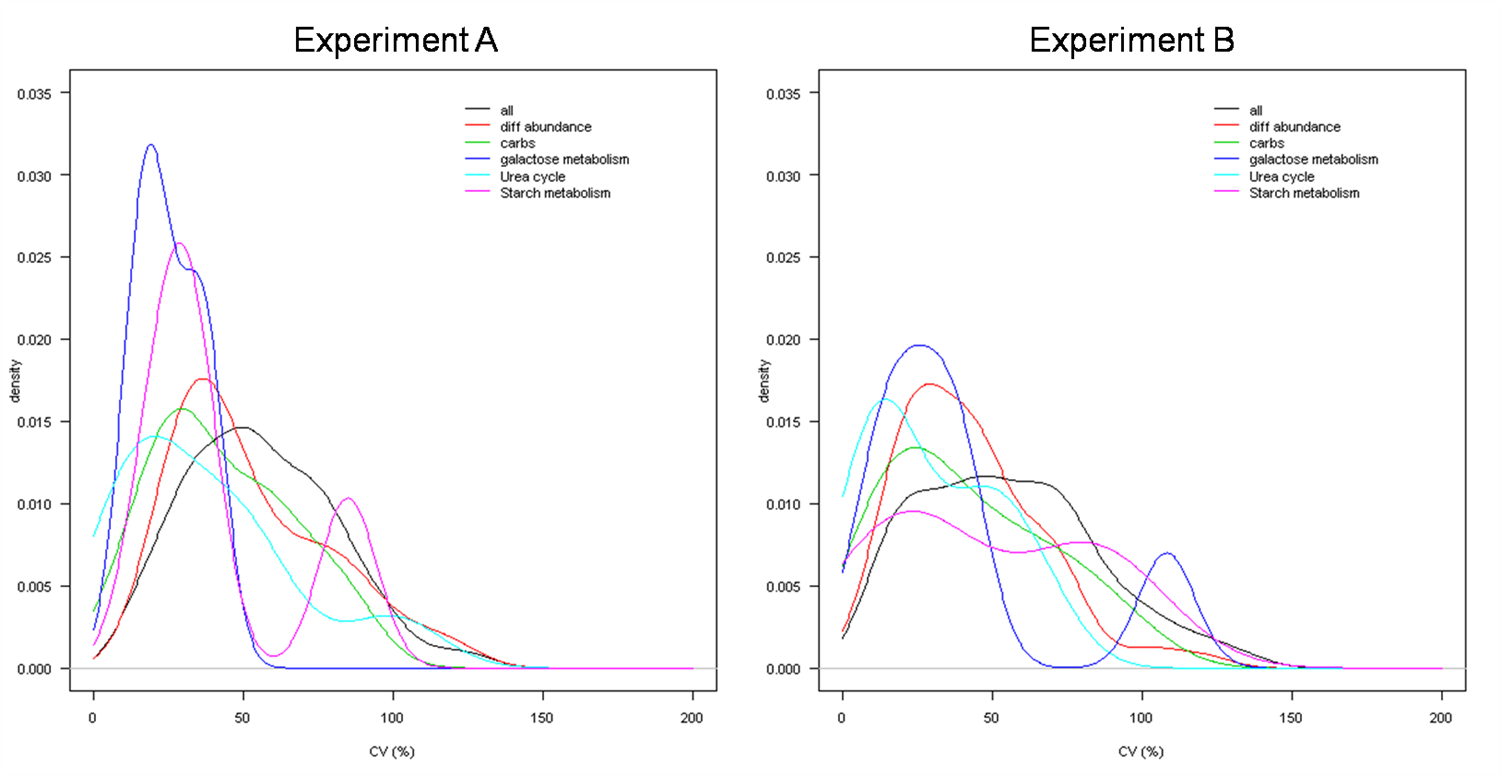

Supplement: Figure S5 — Distributions of coefficients of variations (CV) of subsets of metabolites across 96 Arabidopsis accessions in the AM (left) and PM (right) experiments. Compared are all detected metabolites (black), metabolites that are differentially abundant between experiments (red), carbohydrates (green), metabolites involved in galactose metabolism, KEGG ID: ath00052 (blue), metabolites involved in the urea cycle, KEGG ID: ath00220 (cyan), and metabolites involved in starch and sucrose metabolism, KEGG ID: ath00500 (pink). (0.26 MB TIF) [file pgen.1001198.s007.tif]

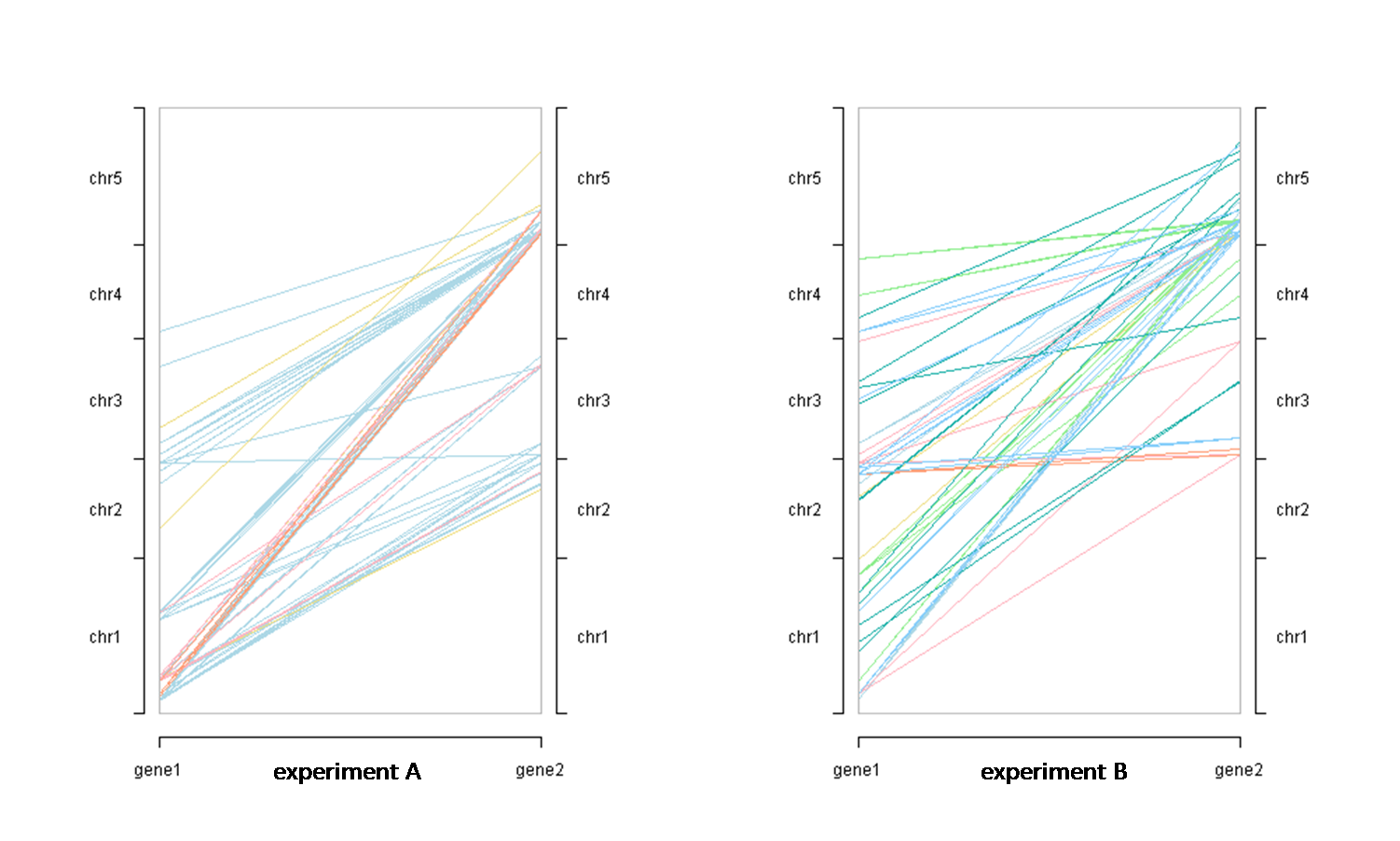

Supplement: Figure S6 — Non-syntenic LD between genes concurrently associated with the same metabolite for the A (left) and B (right) experiments. Shown are gene-pairs (arbitrarily assigned as gene 1 and gene 2) that are (i) associated with the same metabolite, (ii) located on different chromosomes, and (iii) are in strong LD (r2>0.4). Genes 1 and 2 are physically ordered along the two parallel lines. The same colored lines connected the gene-pairs indicate that the genes were associated with the same metabolite. (0.52 MB TIF) [file pgen.1001198.s008.tif]

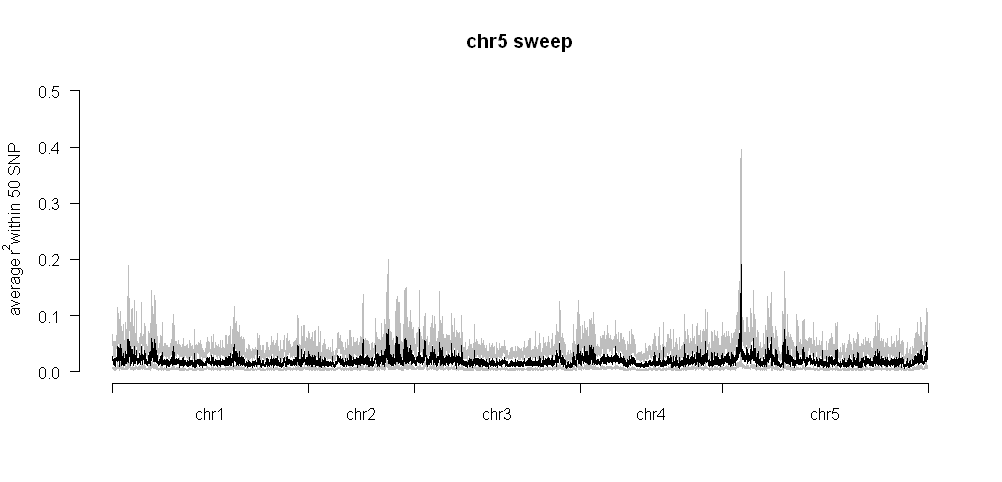

Supplement: Figure S7 — LD between the Chr5 recent positive selective sweep and the genome. r2 was calculated for all 219 SNP within the sweep region of Chr5:2,790,000-2,900,000 against all of ∼ 250,000 available SNP across the genome. The sliding averages of the medians (black) and the 25th and 75th percentile (grey lines) in 50 SNP-intervals are plotted. (0.05 MB TIF) [file pgen.1001198.s009.tif]

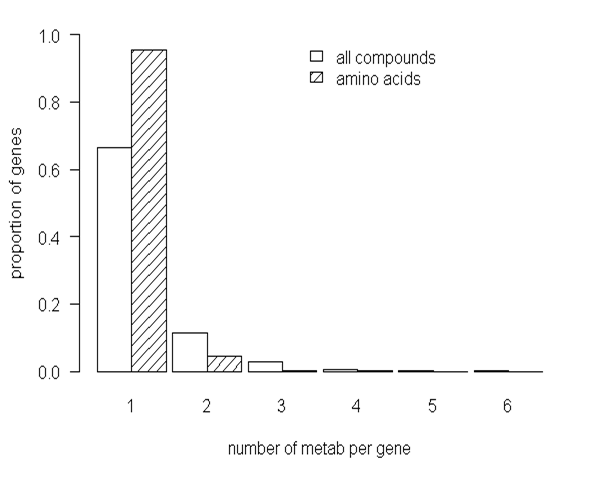

Supplement: Figure S8 — Significant gene association rate with amino acids. Proportion of the genes showing significant association with the corresponding numbers (x-axis) of metabolites for all 266 metabolites (white) and for 20 amino acids (hashed bars). (0.05 MB TIF) [file pgen.1001198.s010.tif]

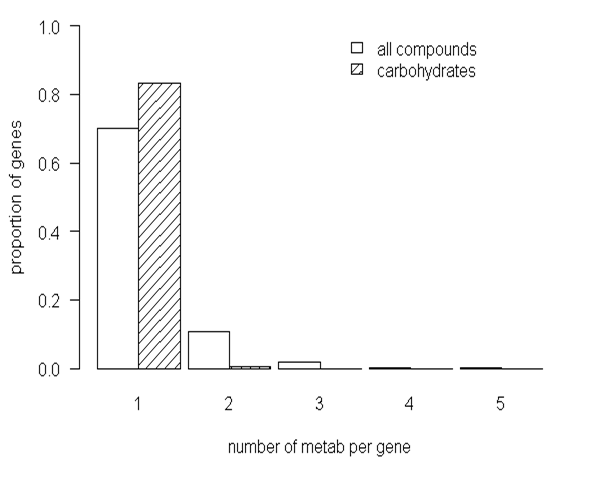

Supplement: Figure S9 — Significant gene association rate to carbohydrates. Proportions of genes significantly associated with the corresponding numbers (x-axis) of metabolites for all 255 metabolites (white bars) and for 15 carbohydrates (shaded bars). (0.05 MB TIF) [file pgen.1001198.s011.tif]
